# Filling the gap between the heart and the body in acromegaly: a case-control study

**DOI:** 10.1007/s12020-022-03232-3

**Published:** 2022-10-30

**Authors:** Ivana Ságová, Milan Dragula, Marián Mokáň, Peter Vaňuga

**Affiliations:** 1 Department of Endocrinology, National Institute of Endocrinology and Diabetology, Ľubochňa, Slovakia; 2 Comenius University Jessenius Faculty of Medicine, 1st Department of Internal Medicine, University Hospital Martin, Martin, Slovakia; 3 Cardiology clinic University Hospital Martin, Martin, Slovakia

**Keywords:** Acromegaly, Dual-energy X-ray absorptiometry, Echocardiography, Insulin-like growth factor 1

## Abstract

**Objective:**

Cardiovascul diseases are the most common comorbidities in acromegaly. Potential parameters in pathology of cardiovascular comorbidities are changes in levels of growth hormone (GH) and insulin-like growth factor 1 (IGF-1) as well as body composition parameters.

**Purpose:**

The aim of this study was to examine morphological and functional parameters of the cardiovascular system by echocardiography and to assess its relationship with disease activity and body composition parameters.

**Methods:**

We prospectively enroled 129 acromegalic patients (82 females, 47 males) and 80 healthy controls (53 females, 27 males) matched for age, gender, and BMI. All patients underwent two-dimensional echocardiography. Body composition parameters were assessed by dual-energy X-ray absorptiometry.

**Results:**

Acromegaly patients presented with higher left ventricle mass (LVM) compared to controls (LVMI: 123 ± 45 g/m^2^ vs 83 ± 16 g/m^2^, *P* < 0.001). Prevalence of left ventricle hypertrophy in acromegaly patients was 67% (78% concentric, 22% eccentric). IGF -1 levels, BMI, and lean mass positively correlated with LVM in all acromegaly patients (*P* < 0.001). Fat mass positively correlated with LVM in females (*R* = 0.306, *P* = 0.005), but this correlation was not found in males. We did not find any difference in size of the left and right ventricle between acromegaly patients and controls. Acromegaly patients presented with left atrium enlargement, diastolic dysfunction and low incidence of systolic dysfunction. Valvopathy was found in 43% of patients with predominant (31%) prevalence of mitral regurgitation.

**Conclusion:**

Our study demonstrates higher prevalence of cardiovascular comorbidities in acromegaly patients and the impact of IGF-1 levels and body composition parameters in pathology in some of these comorbidities.

## Introduction

Acromegaly is a rare chronic disease, characterised by overproduction of growth hormone (GH), mainly caused by anterior pituitary tumours. Long-term presence of elevated GH and insulin-like growth factor 1 (IGF-1) levels accompanying this disease is associated with cardiovascular, rheumatologic, pulmonary and metabolic complications. Cardiovascular diseases (CVD) are the most common comorbidities in patients with acromegaly. They include arterial hypertension, atherosclerosis, coronary heart disease, septal hypertrophy, left ventricular dysfunction, valvopathy, arrhythmias, and endothelial dysfunction. In addition, specific acromegalic biventricular cardiomyopathy has also been reported in acromegaly [1]. In the pathogenesis of CVD, GH and IGF-1 act directly on the myocardium, where they cause left ventricular hypertrophy, increase myocardial contractility and influence calcium influx in cardiomyocytes. GH and IGF-1 also have an indirect effect on the heart by regulating peripheral vessel resistance [2]. The main predisposing factors for developing arterial hypertension (AH) and cardiac abnormalities in acromegaly are duration of GH hypersecretion, age and body mass index (BMI) [2]. Cardiovascular comorbidities in acromegaly patients significantly increase morbidity and mortality. Frequent concomitant diseases (impaired glucose metabolism, dyslipidaemia, sleep apnoea syndrome) also contribute to the increased mortality from CVD diseases in acromegaly. Due to the subtle progress of acromegaly, the diagnosis is often delayed for up to 8–10 years after the onset of clinical manifestations, which means that patients are rarely diagnosed before the age of 40 [3]. Effective specific treatment of acromegaly has a significant effect on the improvement of CVD with the possibility of recovery to normal, especially in young patients with short duration of acromegaly [4].

This study had two main aims. One was to analyse morphological and functional parameters of the cardiovascular system based on echocardiography in acromegaly patients compared with sex, age and BMI matched controls. The other was to establish any possible relationships between morphological and functional parameters of the cardiovascular system and 1) the disease activity and 2) body composition parameters, based on Dual-Energy X-ray Absorptiometry (DXA). Secondary aims were to determine prevalence of arterial hypertension and valvopathy in acromegaly patients.

## Materials and methods

This prospective cross-sectional study was performed at the National Institute of Endocrinology and Diabetology in Ľubochňa from June 2016 to February 2022. The study protocol was approved by the regional medical ethics committee (EK NEDÚ n.o.). Written informed consent was obtained from all participants after all procedures had been fully explained.

### Patients

We prospectively examined 129 acromegaly patients (82 females, 47 males) and 80 healthy volunteers (53 females, 27 males) matched for sex, age and BMI, who served as a control group.

Acromegaly patients:

- The inclusion criterion for acromegaly patients was presence of acromegaly. The diagnosis was based on established criteria: GH levels after oral glucose tolerance test (oGTT) > 1µg/l and IGF-1 levels above normal range for age and sex [5].
- The duration of acromegaly was estimated from the patient´s past photographs and the onset of clinical symptoms/findings of acromegaly.

Healthy age-, gender- and BMI-matched control subjects:

- Subjects without presence of acromegaly (normal IGF-1 values)
- Other exclusion criteria for healthy subjects were as follows: presence of coronary artery disease, history of stroke/transient ischaemic attack, pulmonary embolism, LVEF < 50%, presence of diabetes mellitus (DM), chronic obstructive pulmonary disease, or history of cancer.

This study included 129 acromegaly patients, 27 of them were newly diagnosed, without a treatment of acromegaly. 102 of the patients with acromegaly diagnosis lasting from 1 year to several years received a previous treatment of acromegaly. 85 of the patients underwent transsphenoidal surgery, 36 of them received primary medical treatment with somatostatin analogues (SSA) before the surgery. The surgery cured 28 patients. 57 patients with not-radical transsphenoidal surgery underwent post-surgery treatment either with only SSA (40 patients), in combination with pegvisomant (15 patients) or in combination with cabergoline (2 patients). 17 patients did not undergo the surgery due to contraindication to surgical treatment or location of the pituitary tumour. They were treated primarily with SSA and continued this single treatment (11patients) or the treatment in combination with pegvisomant (6 patients). Radiotherapy was used adjunctively in 41 patients with tumour remnants and persistently active disease that did not respond to post-surgery pharmacological therapy.

For statistical analyses, all acromegaly patients (AP) were divided into 3 subgroups. One consisted of newly diagnosed acromegaly patients (nAP), without any treatment of acromegaly. Acromegaly patients with previous treatment were divided into controlled acromegaly patients (cAP) and uncontrolled acromegaly patients (uAP) based on disease control. Uncontrolled disease was defined as IGF-1 level above upper limit of normal reference range for age, sex or lack of GH suppression during oGTT. Otherwise, the disease was considered controlled, even when it was maintained by ongoing treatment.

We collected data regarding the presence of cardiovascular risk factors and recorded the presence of comorbid conditions including AH (diagnosis of AH or use of hypertensive medication) and DM (diagnosis of DM or use of antihyperglycemic medication).

### Clinical examination

In all study subjects, we performed anthropometric measurements including weight (kg) and height (cm). BMI was calculated as weight in kilograms divided by square of height in metres (kg/m^2^). Body surface area (BSA) was calculated using Mosteller’s formula BSA = (((height in cm) × (weight in kg))/3600)^½^ [6]. Blood pressure was measured after a 5-minute rest using Omron M5-I (Omron Health care Europe BV, Hoofddorp, Netherlands). Arterial hypertension (AH) was diagnosed according to the guidelines of the European Society of Hypertension [7].

### Laboratory examinations

All subjects underwent the following blood tests: GH, IGF-1, pituitary hormones, blood count, creatinine concentration, liver enzymes, serum lipid profile, serum fasting glucose, insulin, and glycated haemoglobin. Glucose metabolism disorders were diagnosed according to American Diabetes Association Guidelines [8]. Venous blood samples were obtained between 07:00 and 08:00 am after overnight fasting. IGF-1 and GH levels were measured using a chemiluminescent immunometric assay ECLIA (Immulite 2000 assay, Siemens Healthcare Diagnostics Products Ltd., United Kingdom). Intraassay variability (CV) was for IGF-1 between 3.0–7.6% and for GH between 6.5–6.6%. Normal level for serum GH was 5 ng/ml. Normal range of IGF-1 was adjusted for sex and age.

### Echocardiography examination

All patients underwent two-dimensional echocardiography using Vivid 9 (Horten, Norway). Echocardiography was performed using all standard views. All measurements of left atrium (LA) and left ventricle (LV) were made in accordance with guidelines of the European Society of Echocardiography [9]. We measured LV diastolic diameter and myocardial thickness: posterior wall (PW) and interventricular septum (IVS). Left ventricle mass (LVM) was calculated using Deveroux and Reichek Cube Formula [10]. LVM was indexed to BSA. LV hypertrophy was defined as left ventricle mass index (LVMI) > 115 g/m^2^ in men and >95 g/m^2^ in women. Relative wall thickness (RWT) was calculated as 2 times posterior wall diastolic diameter (PWDd) divided by left ventricular end-diastolic parameter (LVEDd) [9]. Increased LVM with RWT > 0.42 cm is consistent with concentric LV hypertrophy while RWT < 0.42 cm is consistent with eccentric hypertrophy. Ejection fraction (EF) was estimated by Simpson´s biplane method. According to the guidelines the low normal level was set at EF = 55%. LA indexed volume (LAVI) was measured by area-length method using the apical four-chamber and two-chamber view. Diastolic dysfunction was assessed in the apical 4 chamber view by measurement of peak velocities of early (E) and late diastolic flow (A), pulsed early diastolic waves (E´), and E/A and E/E´ ratios, using Tissue Doppler imaging. Diastolic dysfunction was graded from I to III according to the current guidelines [11]. The right ventricular (RV) diameters were measured in the four-chamber apical view. TAPSE was recorded as the peak excursion of the lateral tricuspid annulus by M-mode echocardiography. Valve disease was considered to be present if any functional defect (stenosis or regurgitation) other than physiological trace was reported. We defined non-significant valve disease severity as Grade I (mild) and significant valve disease severity as Grades II to IV (II - moderate, III - moderate-to-severe, IV- severe).

### Dual-energy X-ray absorptiometry

Body composition (fat mass and lean mass) was determined by DXA (Hologic Horizon A, Bedford, MA) using whole-body software version 13.6. Coefficient of variation was 0.78% for fat mass and 0.52% for lean mass. All patients underwent DXA.

### Statistical analyses

All statistical analyses were performed using IBM SPSS version 25 (IBM SPSS Statistics, IBM Corporation, IL, USA). Statistical power was calculated using the G*Power v. 3.0.1 software. Continuous data are presented as mean and ±standard deviation (SD). Categorical data are presented as numbers and percentage. Inter-group comparisons were performed using either Student’s *t* tests or Mann–Whitney tests, depending upon the normality distribution of the studied parameter. In each statistical test performed, the criteria for statistical significance was *p* ≤ 0.05. All tests were two-tailed.

## Results

A total of 129 acromegaly patients (82 females, 47 males) were included in the study. The mean age of AP at the time of entering the study was 55 ± 12 years. The age-, sex- and BMI-matched controls (CON) consisted of 80 healthy subjects (53 females, 27 males) with the mean age of 56 years ±10 years. Baseline characteristics of all subjects are summarised in Table 1.

**Table 1:** Baseline characteristics of subjects

Characteristics	Acromegaly patients (*n* = 129)	Healthy controls (*n* = 80)	Newly diagnosed acromegaly patients (*n* = 27)	Uncontrolled acromegaly (*n* = 53)	Controlled acromegaly (*n* = 49)	Acromegaly patients vs. healthy controls *P*-value	Groups comparison *P*-value
Sex (M/F)	47/82	27/53	12/15	20/33	15/34	–	–
Disease duration (year)	9 ± 10	–	2 ± 3	10 ± 9	13 ± 11	–	<0.001^a,b^ NS^d^
Age at the time of study (year)	55 ± 12	56 ± 10	51 ± 9	55 ± 13	58 ± 12	NS	0.008^b^ 0.013^c^ NS^a,d,e,f^
Baseline GH (ng/ml)	3.54 ± 6.23	0.27 ± 0.19	9.58 ± 9.28	2.66 ± 5.19	1.17 ± 0.89	<0.001	<0.001^a,b,c,e,f^ 0.045^d^
IGF-1 (ng/ml)	308 ± 247	135 ± 39	627 ± 282	310 ± 161	130 ± 42	<0.001	<0.001 ^a,b,c,d,e^ NS^f^
Creatinine (µmoll/l)	72 ± 20	73 ± 15	67 ± 24	71 ± 20	75 ± 16	NS	NS^a,b,c,d,e,f^
Arterial hypertension (%)	57%	19%	52%	58%	59%	<0.001	<0.001^c,e,f^ NS^a,b,d^
Systolic BP(mmHg)	123.1 ± 9.2	122.4 ± 11.7	123.9 ± 13.9	123.5 ± 12.4	122.9 ± 13.2	NS	NS^a,b,c,d,e,f^
Diastolic BP(mmHg)	77.8 ± 8.9	77.1 ± 9.7	78.3 ± 8.9	78.2 ± 9.8	77.9 ± 10.3	NS	NS^a,b,c,d,e,f^
DM (%)	19%	0%	15%	25%	14%	<0.001	<0.001^c,e,f^ NS^a,b,d^
Dyslipidaemia (%)	59%	33%	44%	68%	57%	< 0.001	<0.001^e,f^ 0.040^c^ 0.043^a^ NS^b,d^
Smoking history (%)	19%	15%	22%	15%	20%	NS	NS^a,b,c,d,e,f^
BSA (m^2^)	1.99 ± 0.24	1.93 ± 0.23	2.05 ± 0.29	2.01 ± 0.22	1.97 ± 0.23	NS	0.025^c^ NS^a,b,d,e,f^
BMI (kg/m^2^)	30 ± 6	30 ± 5	31 ± 6	31 ± 6	29.9 ± 5	NS	NS^a,b,c,d,e.f^
Fat mass (kg)	32 ± 9	32 ± 10	31 ± 10	32 ± 10	31 ± 9	NS	NS^a,b,c,d,e,f^
Lean mass (kg)	57 ± 15	51 ± 12	64 ± 19	58 ± 13	51 ± 13	0.003	0.002^b^ < 0.001^c^ 0.007^d^ 0.002^e^ NS^a,f^

Data is presented as mean ±standard deviation (SD) and as percentage

Level of significance was set at *p ≤ 0.05

*GH* growth hormone, *IGF-1* insulin-like-growth factor 1, *BMI* body mass index, *BSA* body surface area

^a^Newly diagnosed acromegaly patients vs. uncontrolled

^b^Newly diagnosed acromegaly patients vs. controlled

^c^Newly diagnosed acromegaly vs. healthy controls

^d^Uncontrolled vs. controlled

^e^Uncontrolled vs. healthy controls

^f^Controlled vs. healthy controls

### Comparison between acromegaly patients and healthy controls

Mean IGF-1 level in acromegaly patients was 308 ± 247 ng/ml with a GH of 3.54 ± 6.23 ng/ml. Serum levels of GH and IGF-1 were significantly higher in AP compared with CON. There was no statistically significant difference in BSA, BMI, fat mass between AP and CON, but lean mass was higher in AP compared to CON (57 ± 15 kg vs 51 ± 12 kg, *P* = 0.003) (Table 1).

We did not find difference in the size of the left and right ventricles between both groups.

Compared to CON, AP presented with higher LVM (LVMI: 123 ± 45 g/m^2^ vs 83 ± 16 g/m^2^, *P* < 0.001) (Table 2). Prevalence of LV hypertrophy in AP was 67% (78% concentric, 22% eccentric). Significant differences between AP and CON were confirmed in IVS (12.4 ± 2.1 mm vs 10.1 ± 1.0 mm, *P* < 0.001), PWD (11.6 ± 2.0 mm vs 9.5 ± 1.0, *P* < 0.001), and LA size (LAVI: 38.4 ± 7.2 vs 29.1 ± 6.1 *P* < 0.001). There was no statistically significant difference in systolic function between both groups, but we found statistically significant difference in diastolic function measured with E/É and E/A (Table 2).

**Table 2:** Echocardiography parameters

Characteristics	Acromegaly patients (*n* = 129)	Healthy controls (*n* = 80)	Newly diagnosed acromegaly patients (*n* = 27)	Uncontrolled Acromegaly (*n* = 53)	Controlled Acromegaly (*n* = 49)	Acromegaly patients vs. healthy controls *P*-value	Group comparison *P*-value
Ao. Asc. (mm)	33.7 ± 3.4	32.7 ± 2.8	34.4 ± 1.8	33.1 ± 3.8	33.8 ± 3.2	NS	<0.007^c^ NS^a,b,d,e,f^
LAVI (ml/m^2^)	38.4 ± 7.2	29.1 ± 6.1	39.2 ± 6.8	36.9 ± 6.5	32.4 ± 5.2	<0.001	<0.001^c,e^ 0.018^b^ 0.042^d^ NS^a,f^
LVEDd (mm)	50.5 ± 5.8	48.5 ± 2.2	53.7 ± 9.4	51.1 ± 4.0	48.6 ± 3.5	NS	<0.001^c^ 0.006^b^ NS^a,f,d,e^
RVEDd (mm)	32.8 ± 3.8	31.4 ± 2.3	31.2 ± 3.9	31.7 ± 2.6	32.4 ± 2.9	NS	NS
IVSDd (mm)	12.4 ± 2.1	10.1 ± 1.0	13.5 ± 2.3	13.2 ± 1.7	10.9 ± 1.2	<0.001	<0.001^b,c,d,e,f^ NS^a^
PWDd (mm)	11.6 ± 2.0	9.5 ± 1.0	13 ± 2.5	12.3 ± 1.5	10.2 ± 1.1	<0.001	<0.001^b,c,d,e,f^ NS^a^
LVM (g)	248 ± 108	159 ± 28	324 ± 174	268 ± 71	185 ± 37	<0.001	<0.001^b,c,d,e,f^ NS^a^
LVMI (g/m^2^)	123 ± 45	83 ± 16	154 ± 72	133 ± 29	96 ± 19	<0.001	<0.001^b,c,d,e,f^ NS^a^
LVH (%)	67%	18%	81%	83%	43%	<0.001	<0.00^b,c,d,e,f^ NS^a^
RWT > 0.42 (%)	78	35	93	89	73	<0.001	<0.001^b,c,d,e^ 0.039^f^ NS^a^
LVEF (%)	58 ± 6	57 ± 3	54 ± 10	58 ± 5	59 ± 3	NS	0.039^b^ NS^a,c,d,e,f^
E/E´	10.9 ± 3.62	9.2 ± 3.11	11.0 ± .5.46	10.4 ± .4.35	9.6 ± 3.94	0.003	0.012^c^ 0.029^b^ 0.038^e^ NS^a,d,f^
E/A	0.95 ± 0.4	1.15 ± 0.3	0.9 ± 0.35	1.0 ± 0.3	1.10 ± 0.5	0.001	0.008^c^ 0.027^b^ NS^a,d,e,f^
TAPSE (mm)	22.2 ± 2.9	22.5 ± 1.1	22.3 ± 2.6	22.1 ± 3.2	22.3 ± 2.7	NS	NS
Valvopathy (%)	43	10	67	38	35	<0.001	<0.001^c,e,f^ 0.007^b^ 0.014^a^ NS^d^

Data is presented as mean ±standard deviation (SD) and as percentage

Level of significance was set at *p ≤ 0.05

*Ao. Asc.* ascending aorta, *LAVI* left atrial volume index, *LVEDd* left ventricular end-diastolic parameter, *RVEDd* right ventricular end-diastolic parameter, *IVSDd* interventricular septum diastolic diameter, *PWDd* posterior wall diastolic diameter, *LVM* left ventricular mass, *LVMI* left ventricular mass index, *LVH* left ventricle hypertrophy, *RWT* relative wall thickness, *LVEF* left ventricular ejection fraction, *E/E´* mitral E-wave velocity divided by mitral annular velocity, *E/A* mitral E-wave divided by mitral A-wave velocity, *TAPSE* tricuspid annular plane systolic excursion

^a^Newly diagnosed acromegaly patients vs. uncontrolled

^b^Newly diagnosed acromegaly patients vs. controlled

^c^Newly diagnosed acromegaly vs. healthy controls

^d^Uncontrolled vs. controlled

^e^Uncontrolled vs. healthy controls

^f^Controlled vs. healthy controls

### Prevalence of arterial hypertension, valvopathy and dyslipidaemia in acromegaly patients

AP had higher prevalence of AH compared to CON (57 vs 19%, *P* < 0.001). There was no statistically significant difference in diastolic and systolic BP, in both groups; BP was well controlled (Table 1). The prevalence of valvopathy was higher in AP compared to CON (43% vs 10%) (Table 2). Significant valvopathy (moderate to severe) was found in 4% of AP. We found higher prevalence of dyslipidaemia in AP compared to CON (59% vs 33%) (Table 2). The prevalence of DM was 19%. We cannot compare it with CON, because of the exclusion criteria.

### Comparison between subgroups of acromegaly patients

Newly diagnosed acromegaly patients were presented with the highest IGF-1 and GH levels compared to uAP and cAP (*P* < 0.001). There was no difference in BMI, BSA, and fat mass between the subgroups. Lean mass was significantly higher in nAP and uAP compared to cAP (Table 1). There was no statistically significant difference in prevalence of AH and DM between the subgroups. Dyslipidaemia was more presented in uAP compared to the other subgroups.

**Fig. 1:**
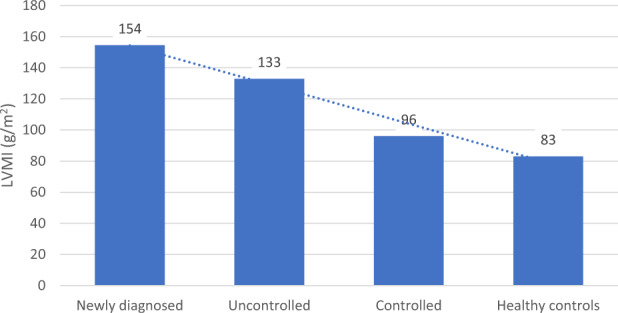
Comparison of LVMI between subgroups of acromegaly patients and healthy controls. LVMI: left ventricular mass index

We did not find any difference in the size of RV between the subgroups, but LV was larger in nAP compared to cAP (Table 2). LVM was significantly higher in nAP and uAP compared to cAP with higher prevalence of concentric hypertrophy (Fig. 1). LA size was higher in nAP and uAP compared to cAP (Table 2). There was no statistically significant difference in IVS, PWD as well as in systolic function between the subgroups. In nAP and uAP, diastolic function was impaired compared to cAP. Valvopathy was more presented in nAP compared to the other subgroups (Table 2).

### Relationships between activity of acromegaly, body composition parameters and echocardiography parameters in acromegaly patients

We observed a trend to positive correlation between IGF-1 and BMI in AP (*R* = 0.256, *P* = 0.077). Positive correlation was found between IGF-1 levels and BSA in the whole AP group (*R* = 0.233, *P* < 0.001) and in both sex subgroups. IGF-1 positively correlated with lean mass in AP (*R* = 0.401, *P* < 0.001), in males (APm) (*R* = 0.487, *P* < 0.001) and in females (APf) (*R* = 0.235, *P* = 0.006). No correlation was observed between IGF-1 levels and fat mass in AP (Table 3).

**Table 3:** Correlation analyses

	Lean mass			Fat mass			Lean mass			Fat mass			Lean mass			Fat mass		
	Acromegaly patients (*n* = 129) *P*-value	Females (*n* = 82) *P*-value	Males (*n* = 47) *P*-value	Acromegaly patients (*n* = 129) *P*-value	Females (*n* = 82) *P*-value	Males (*n* = 47) *P*-value	Newly diagnosed acromegaly patients (*n* = 27) *P*-value	Females (*n* = 15) *P*-value	Males (*n* = 12) *P*-value	Newly diagnosed acromegaly patients (*n* = 27) *P*-value	Females (*n* = 15) *P*-value	Males (*n* = 12) *P*-value	Uncontrolled Acromegaly (*n* = 53) *P*-value	Females (*n* = 33) *P*-value	Males (*n* = 20) *P*-value	Uncontrolled Acromegaly (*n* = 53) *P*-value	Females (*n* = 33) *P*-value	Males (*n* = 20) *P*-value
IGF-1 (ng/ml)	**<0.001**	**0.006**	<**0.001**	0.338	0.229	0.758	**0.042**	0.898	0.947	0.201	0.409	0.303	**0.015**	0.945	**0.028**	0.757	0.632	0.848
LVEDd (mm)	**<0.001**	<**0.001**	<**0.001**	**0.004**	<**0.001**	0.247	<**0.001**	0.118	0.035	0.112	0.201	0.215	<**0.001**	0.146	**0.018**	**0.028**	**0.049**	0.076
IVSDd (mm)	**<0.001**	<**0.001**	**0.002**	**0.032**	**0.005**	0.532	**0.004**	0.280	0.571	0.409	0.310	0.796	**0.002**	<**0.001**	**0.05**	**0.030**	**0.007**	0.487
PWDd (mm)	**<0.001**	**< 0.001**	<**0.001**	<**0.001**	**0.041**	0.568	**0.041**	0.512	0.358	0.448	0.483	0.762	**0.012**	**0.009**	**0.008**	0.110	**0.05**	0.674
LVM (g)	**<0.001**	**< 0.001**	<**0.001**	**0.030**	**0.005**	0.183	**0.003**	0.333	0.05	0.157	0.282	0.271	<**0.001**	**0.002**	**0.015**	**0.014**	**0.003**	0.225
LVEF (%)	**0.016**	0.165	0.12	**0.03**	0.301	0.239	**0.034**	0.563	0.270	0.230	0.364	0.327	0.904	0.356	0.835	0.453	0.728	0.359

Bold values show the level of significance set at *p* ≤ 0.05

*IGF-1* insulin like growth factor −1, *BMI* body mass index, *LVEDd* left ventricular end-diastolic parameter, *IVSDd* interventricular septum diastolic diameter, *PWDd* posterior wall diastolic diameter, *LVM* left ventricular mass, *LVMI* left ventricular mass index, *LVEF* left ventricular ejection fraction

IGF-1 levels positively correlated with LVM in AP (*R* = 0.575, *P* < 0.001); APm (*R* = 0.621, *P* < 0.001) and APf (*R* = 0.465, *P* < 0.001). We did not find positive correlation between duration of acromegaly and LVM. Positive correlation was found between BSA and LVM in AP (*R* = 0.394, *P* < 0.001), APm (*R* = 0.456, *P* < 0.001) and APf (*R* = 0.337, *P* = 0.002). BMI positively correlated with LVM in AP (*R* = 0.484, *P* < 0.001), APm (*R* = 0.381, *P* = 0.008) and APf (*R* = 0.447, *P* < 0.001).

Lean mass positively correlated with LVM in AP (*R* = 0.561, *P* < 0.001), APm (*R* = 0.606, *P* < 0.001) and APf (*R* = 0.422, *P* < 0.001) (Table 3). Fat mass positively correlated with LVM in AP (*R* = 0.240, *P* = 0.030), and in APf (*R* = 0.306, *P* = 0.005), but this correlation was not found in APm. Similar results were found in uAP (Table 3).

BSA and lean mass positively correlated with LVED, IVSD, PWD in AP (*P* < 0.001). We observed positive correlations between fat mass and LVED, IVSD, PWD in AP and in APf. No correlation was observed in APm (Table 3).

## Discussion

This prospective cross-sectional study on a large number of acromegaly patients analysed morphological and functional parameters of the cardiovascular system and body composition parameters to determine the mutual relationships. To our knowledge this is the first study investigating the influence of body composition parameters using DXA scan on left ventricular morphology in acromegaly patients.

Cardiovascular diseases are the most common comorbidity in acromegaly [1]. Myocardial hypertrophy may occur prematurely and tends to worsen with disease duration and coexistence of other cardiovascular risk factors [12, 13]. GH/IGF-1 affects 3 main aspects of the cardiovascular system: myocyte growth and structure, cardiac contractility, and vascular function [13, 14]. In addition to stimulating myocyte growth, IGF-1 also promotes collagen synthesis by fibroblasts, while GH increases collagen deposition rate [15]. Acromegalic cardiomyopathy develops in three stages; 1) the early phase of asymptomatic left ventricle hypertrophy (LVH) and increased systolic output, 2) the middle phase with evident LVH, diastolic dysfunction and decreased systolic output at exercise, and 3) the end-stage dilated cardiomyopathy with heart failure and systolic dysfunction at rest [15, 16].

Biventricular hypertrophy is characteristic for acromegaly [12]. We did not find difference in the size of the left and right ventricles between acromegaly patients and controls, which is in agreement with other studies [17–19]. The results indicate that there is no difference in LV size, RV size, and RV free-wall thickness between AP and controls. Only a few studies have focused on the size of RV in acromegaly. A small study of acromegaly patients (*n* = 20) reported abnormal thickness of RV free wall [20, 21]. However, another study on 108 AP did not confirm a difference in diastolic RV dimension compared to controls [22]. The influence of GH / IGF-1 on the morphology of RV is uncertain [17].

Reported incidence of left myocardial hypertrophy in acromegaly patients in different studies varies between 10 and 80% [18, 23–35]. The difference could be caused by different definitions of left myocardial hypertrophy, research designs (retrospective, prospective or cross-sectional), and investigative methods (MR/echocardiography) used in the studies. In medical practice, two-dimensional (2D) echocardiography is the most common method for measuring of LV mass. However, this parameter is influenced by weight and height and is commonly indexed to BSA such as LVMI. Our study found increased LVM and LVMI in AP compared to controls. The prevalence of LV hypertrophy in AP was 67%, of which 78% had concentric hypertrophy and 22% eccentric hypertrophy. This suggests that hemodynamic determinant of pressure overload (increased afterload and systolic stress, like in hypertension) is more frequent than volume overload (increased preload and diastolic stress) [36].

Risk factors for myocardial hypertrophy in patients with acromegaly are still unclear. One retrospective study analysing cardiovascular-related complications of 205 newly diagnosed patients with active acromegaly confirmed duration of acromegaly as the main risk factor for cardiomyopathy [37]. A study on 108 acromegaly patients found age and increased BMI to be risk factors for acromegaly cardiomyopathy [22]. In addition, a multivariate analysis showed that AH and IGF-1 levels were determinants of LV hypertrophy in acromegaly [23].

In our study, left ventricle mass depended on IGF-1 levels and but not on the duration of the disease. Greater LVM did not depend on AH (no significant difference in prevalence of AH or BP in subgroups). Our findings show the importance of IGF-1 in pathogenesis of left ventricle hypertrophy in acromegaly.

We also investigated the influence of body composition parameters on LV morphology. The common practice of echocardiography, indexing is based on the linear relationship between the body size and LVM. Patients with larger body size are expected to have greater LVM. Some studies on healthy adults have shown that lean body mass correlates well with LVM [38, 39]. The effect of fat mass on LVM is still uncertain. Previously, obesity was associated with eccentric LV hypertrophy because of obesity-related volume overload [40]. In contrast, recent echocardiography studies show that both LV cavity size and wall thickness may be increased in obese subjects with wall thickness increased more than cavity size (concentric LV remodelling without a change in EF) [41, 42].

Our acromegaly patients presented with higher lean mass, with no other differences in body composition parameters (BMI, BSA, fat mass) compared to controls. Positive correlations between BMI and BSA with LVM were found in AP, in both sexes. More recent data in healthy adults have demonstrated that increased BMI is strongly related to increasing LV mass independent of AH [43]. In a study on AP, Guo et al. found increased BMI to be an independent risk factor also in acromegaly cardiomyopathy [22]. In healthy adults, lean and fat mass tends to increase together with increasing of BMI [44]. However, in active acromegaly, fat mass decreases and lean mass increases [45, 46]. These changes correlate with severity of the disease and GH/IGF-1 levels, and tend to normalise after successful treatment [47].

We found a significant correlation between lean mass measured by DXA and LVM in all acromegaly patients in both sexes. Interestingly, fat mass positively correlated with LVM in females while in males this correlation was not observed. When divided into subgroups, the correlation in female subgroup was found in with uncontrolled acromegaly patients. It was not found in controlled subgroup, in whom body composition is expected to be similar to that of healthy population. No correlation in newly diagnosed acromegaly females may be caused by the small size of the group. The importance of adipose mass on variability of LVM in females is as high as that of AH [39]. Lean mass is independently related to LVM in both sexes. The “cardiac steatosis” has functional consequences in females but not in males [48]. Although concentric hypertrophy is predominant in both sexes, females show a stronger association between fat mass and a regional increase in cardiac volume [49]. This difference between the sexes could be caused by the mechanism of how body fat is stored and the consequent haemodynamic effect. Visceral distribution of body fat, more common in males, is associated with concentric LV remodelling [49]. Peripheral distribution of body fat, more common in females, is associated with eccentric LV remodelling [50]. These morphological adaptations are independent of systolic BP and suggest that changes in both preload and afterload are influential in determining local patterns of remodelling [51].

In our study, acromegaly patients presented with more frequent occurrence of left atrium enlargement, diastolic dysfunction and low incidence of systolic dysfunction. It is unclear whether increased LA volume results directly from LV hypertrophy and impaired diastolic function, or increased expression of GH receptors in cardiomyocytes of LA [17]. The most common reported changes in acromegaly patients are LV hypertrophy and impaired diastolic function [17]. We found diastolic dysfunction in 62.8% AP, in 91.5% of them Grade I diastolic dysfunction was present. Only 4 from all AP had systolic dysfunction (LVSD) with EF < 50% (1 female, 3 males). Two of them had EF > 40%. Two male patients were diagnosed with severe LVSD and exhibited remarkably decreased EF (22% and 29%). LV hypertrophy causes diastolic and more rarely systolic dysfunction. Occurrence of LV systolic dysfunction in AP has been observed with long-standing active disease [37]. Incidence of diastolic dysfunction in AP was between 11.3 and 100%, with the average incidence of 46.3% [18, 52–55]. Diastolic dysfunction is characterised by an inadequate filling capacity [56] and can be reversed by medical treatment and LVM reduction [37]. Although diastolic dysfunction is frequently observed in acromegaly, it is usually mild, with no clinical consequences, and the progression to systolic dysfunction has rarely been described in more recent studies [17, 35].

Valvopathy is common in acromegaly cardiomyopathy, affecting 75% of patients at the time of diagnosis [57]. In the pathophysiology of valvopathy, GH/IGF-1 directly effects connective tissue. Degeneration of valve tissue interstitium causes aortic and mitral insufficiency [58]. A study of 40 acromegaly patients echocardiographically confirmed a 30% incidence of aortic regurgitation (AoR) and a 5% incidence of mitral regurgitation (MiR) [58]. An independent risk factor for valvopathy is the duration of acromegaly [59]. A prospective study on 18 uncontrolled AP found an increasing incidence of MiR during the follow-up; at baseline, 39% of patients had MiR, which increased to 78% after an average of 1.9 years of the follow-up [60].

We also found higher incidence of valvopathy in acromegaly patients compared to controls. However, in contrast with previous studies, we observed 31% incidence of MiR and 11% incidence of AoR. Only 4% of acromegaly patients had significant valvopathy (moderate to severe).

## Conclusion

In our study, we confirmed higher incidence of left ventricle hypertrophy in acromegaly patients compared to healthy controls. We found left ventricle hypertrophy to depend on IGF-1 levels but not on the duration of the disease. Lean body mass strongly correlated with left ventricular mass in both sexes. Our results suggest that, in acromegaly females, left ventricle hypertrophy could also be associated with fat mass. We did not find a statistically significant difference in the size of either the left or the right ventricle in acromegaly patients compared to healthy controls. Acromegaly patients had more frequent occurrence of left atrium enlargement, diastolic dysfunction, and low incidence of systolic dysfunction than healthy controls. Valve regurgitation was common in acromegaly patients with predominance of mitral regurgitation.

We believe that our results deepened the understanding of pathology of cardiovascular comorbidities in acromegaly and future prospective studies focusing on more relationships between body composition and cardiovascular disorders could be useful in furthering our understanding.

### Strengths and limitations

The strengths of our study are the large number of all acromegaly patients and the implementation of echocardiography and DXA examinations, which enabled us to analyse and compare the impact of multiple factors in a single study. Moreover, this is the first study that investigated (and confirmed) the correlations between lean mass and myocardial hypertrophy in acromegaly patients. The limitation of our study is the relatively small sample size in the subgroups, due to the low disease incidence and the differences in the number of males and females.
